# Can oral squamous cell carcinoma xenografts tumors mirror the original tumor microenvironment? An immunohistochemical analysis

**DOI:** 10.1007/s00428-026-04399-0

**Published:** 2026-01-27

**Authors:** Mateus José Dutra, Brendo Vinicius Rodrigues Louredo, Sebastião Silvério de Sousa-Neto, Hélen Kaline Farias Bezerra, Ana Carolina Prado-Ribeiro, Leandro Luongo Matos, Felipe Martins Silveira, Manoela Domingues Martins, Luiz Paulo Kowalski, Pablo Agustin Vargas, Vivian Petersen Wagner

**Affiliations:** 1 Department of Oral Diagnosis, Piracicaba Dental School, Universidade de Campinas, Piracicaba, SP Brazil; 2 Dental Oncology Service, Instituto Do Câncer Do Estado de São Paulo (ICESP), Faculdade de Medicina da Universidade de São Paulo (FMUSP), São Paulo, Brazil; 3 Head and Neck Service, Instituto Do Câncer Do Estado de São Paulo (ICESP), Faculdade de Medicina, Universidade de São Paulo, São Paulo, Brazil; 4 Department of Diagnosis in Pathology and Oral Medicine, School of Dentistry, Universidad de La República, Montevideo, Uruguay; 5 Department of Oral Pathology, School of Dentistry, Universidade Federal Do Rio Grande Do Sul, Porto Alegre, RS Brazil; 6 Department of Oral Medicine, School of Dentistry, Universidade de São Paulo, Av, Prof. Lineu Prestes, 2227, Cidade Universitária, São Paulo, SP CEP: 05508-000 Brazil

**Keywords:** Squamous cell carcinoma of head and neck, Tumor microenvironment, Biomarkers, T-Lymphocytes, Cancer-associated fibroblasts

## Abstract

This study aimed to characterize the tumor microenvironment (TME) in patient-derived xenograft (PDX) models of oral squamous cell carcinoma (OSCC) and compare histological findings with primary tumors of origin (PTT). OSCC samples from five donor patients were implanted into NOD/SCID mice (PDX0) and subsequently re-implanted into new animals (PDX1), yielding three groups for analysis: PTT, PDX0, and PDX1 (*n* = 5 each). Histological slides with sections were stained with hematoxylin and eosin for grade analysis and subjected to immunohistochemical reactions with antibodies against SMA, CD4, CD8, CD31, CD34, Claudin-1, Vimentin, and Ki-67. Multiple comparisons were performed between samples (PTT, PDX0, and PDX1). The histological grade of PDX0 and PDX1 tumors showed instability across passages. The expression of SMA, claudin-1, vimentin, and Ki-67 was maintained, with no significant differences between PDX0 and PDX1 when compared with PTT. In contrast, the expression of CD4, CD8, CD31, and CD34 was significantly reduced in PDX0 and PDX1 tumors compared with PTT. OSCC PDX tumors may exhibit instability in the degree of differentiation compared with the donor tumors across passages, as well as alterations in certain components of the TME, including cancer-associated fibroblasts, epithelial–mesenchymal transition–related features, and cellular proliferation characteristics.

**Supplementary Information:**

The online version contains supplementary material available at 10.1007/s00428-026-04399-0.

## Introduction

The study of new therapies is substantial for the treatment of different types of cancer; thus, the use of patient-derived xenograft (PDX) models is considered highly valuable in translational cancer research, as well as for understanding molecular pathways of oncogenesis and, especially, in the evaluation of therapies [1, 2].

In the PDX technique, tumor fragments are surgically implanted into immunodeficient mice, where, after successful engraftment, several analyses can be performed [2]. This approach is considered more realistic compared to xenograft tumors derived from cell lines [3]. The PDX technique has already been described as highly relevant for the study of different types of head and neck cancer (HNC) [2], because it preserves key histological features and the tumor microenvironment (TME), providing high accuracy and stability for studies of tumor progression and therapeutic response [4–6], and represents a promising method for investigating other malignant neoplasms in this region.

Oral squamous cell carcinoma (OSCC) accounts for 90% of malignant neoplasms in the oral cavity, making it the most common type in the head and neck region [7, 8]. To investigate carcinogenic events and new therapies for OSCC, different *in vivo* methods have been employed, such as the use of 4-nitroquinoline 1-oxide (4NQO) in preclinical murine models [9–12] or DMBA [13], as well as VX2-induced carcinomas in rabbits [13]. Previous studies have also described the PDX technique as a reliable tool for the study of OSCC [5, 6, 14].

Some studies have reported that PDX models accurately reproduce the histological and immunohistochemical features observed in HNC patients [4, 5]. However, to the best of our knowledge, for OSCC, no prior immunohistochemical study has examined the TME components between PDXs and primary patient tumors (PPT). Therefore, this study aimed to compare histological grade and the immunohistochemical expression of key tumor and TME markers (SMA, Claudin-1, Vimentin, Ki-67, CD4, CD8, CD31, and CD34) between donor tumors and OSCC PDX models.

## Material and methods

### Ethical aspects

This study was approved by the Research Ethics Committee of the Piracicaba Dental School, University of Campinas (FOP/UNICAMP; CAAE: 62896122.0.0000.5418), by the Research Ethics Committee of the Hospital das Clínicas, Faculdade de Medicina da Universidade de São Paulo (HC FM USP; CAAE: 62896122.0.3002.0068), and by the Animal’s Ethical Committee of Hospital das Clínicas, Faculdade de Medicina da Universidade de São Paulo (HC FM USP: protocol number 1777/2022). All procedures followed the principles of the Declaration of Helsinki for human research and the Ethical Guidelines for Animal Experimentation established by the National Council for the Control of Animal Experimentation (CONCEA) [15].

All patients were informed about the objectives of the study and subsequently provided a signed consent form agreeing to participate.

### Patients and collection of tumors samples

Tumor specimens were collected from five male patients (51–69 years old), all smokers, with OSCC of the tongue or floor of the mouth (Supplementary Table 1). The diagnosis was established by incisional biopsy, and all cases were p16-negative. Patients with a history of radiotherapy or chemotherapy, as well as those with human papillomavirus (HPV) associated tumors, were excluded.

Tumor fragments were collected from surgical specimens after resection, ensuring that samples were obtained away from surgical margins to avoid interfering with pathological evaluation. Fragments were also taken from regions free of contamination and unsuitable for engraftment, such as superficial oral mucosa, ulcerated areas, or sites of hemorrhage and necrosis. One fragment was fixed in 10% formalin, processed, and embedded in a paraffin block. The remaining fragments were immediately stored in DMEM culture medium supplemented with 10% fetal bovine serum, penicillin/streptomycin (250 µg/mL) and amphotericin B (2.5 µg/mL) (Nova Biotecnologia, Cotia, SP, Brazil) right after surgery, until rapid implantation into animals (Supplementary Fig. 1).

### Animal surgical procedures and experimental groups

For this study, male NOD/SCID mice aged between 5 and 7 weeks and weighing 20–25 g were used. The animals were housed in microisolators with *ad libitum* access to water and feed under controlled conditions (22 °C and 55% humidity) throughout the *in vivo* experimental phase.

Tumor fragments, measuring approximately 5 × 5 mm, were implanted into the dorsal region of five mice (one fragment per animal), establishing passage 0 of the model (PDX0). After tumor growth in the PDX0 models, the animals were euthanized, and the tumors were collected and divided again into two parts: half of the fragments were fixed in 10% formalin, processed, and embedded in paraffin blocks, and the remaining fragments were implanted into the dorsal region of five new different animals (one fragment per animal), establishing passage 1 of the model (PDX1), following the protocol established by Acasigua et al. [16] and Pearson et al. [2] (Supplementary Fig. 1).

Thus, this study comprised three groups, each composed of patient tumor samples (PTT), model passage 0 (PDX0), and model passage 1 (PDX1), which were compared with each other.

For surgical procedures, the animals were anesthetized with an injectable general anesthetic composed of ketamine (Dopalen, Sespo Ltda, Paulínia, SP, Brazil) and xylazine (Anasedan, Sespo Ltda, Paulínia, SP, Brazil). After achieving an adequate level of anesthesia, antisepsis of the area to be operated on the dorsal region of the animal was performed, followed by a single incision down to the subcutaneous plane (~ 1.5 cm in length), implantation of the tumor fragment, and closure of the wound with 5–0 silk suture thread (Shalon Medical, São Luís de Montes Belos, GO, Brazil). The animals were periodically monitored and evaluated for hair loss or atypical behaviors.

When the tumors reached a volume of approximately 1000 mm^3^, the animals were anesthetized with an injectable general anesthetic composed of ketamine (Dopalen, Sespo Ltda, Paulínia, SP, Brazil) and xylazine (Anasedan, Sespo Ltda, Paulínia, SP, Brazil). An incision was then made, and the tumor was collected. The animals were subsequently euthanized using carbon dioxide inhalation, administered in a gradual form. The entire process was supervised and controlled by a veterinarian. After collection, the tumors were immediately measured, fragmented, fixed in 10% formalin (Lablac, Viçosa, MG, Brazil), then processed and embedded in paraffin blocks.

### Histopathological and immunohistochemical analysis

The histological slides with tissue sections were subjected to staining with hematoxylin and eosin (H&E) and were then analyzed under conventional light microscopy for keratinization grade, nuclear pleomorphism, and mitotic count, according to the classification of Anneroth et al. [17]. The resulting scores were divided into respective grades: 3 to 6, grade I; 7 to 9, grade II; and 10 to 12, grade III.

Immunohistochemical reactions were performed on silanized slides (Starfrost, Knittel, DE) containing 3-µm tissue sections. To assess the immunohistochemical profile of samples, the slides were subjected to immunohistochemical reactions using primary monoclonal antibodies directed against SMA (Dako, Carpinteria, CA, USA) for the evaluation of cancer-associated fibroblasts (CAFs); CD4 (Spring BioScience, Pleasanton, CA, USA) and CD8 (Dako, Carpinteria, CA, USA) for the evaluation of T cells; CD31 (Dako, Carpinteria, CA, USA), and CD34 (Dako, Carpinteria, CA, USA) for the evaluation of blood vessels; Claudin-1 (Diagnostic BioSystems, Pleasanton, CA, USA) and Vimentin (Dako, Carpinteria, CA, USA) for the evaluation of epithelial–mesenchymal transition; and Ki-67 (Dako, Carpinteria, CA, USA) for the evaluation of cell proliferation (Supplementary Fig. 1).

Histological slides were deparaffinized and hydrated before antigen retrieval was performed by heat induction for 15 min in an electric pressure cooker. Endogenous peroxidase activity was blocked with 6% hydrogen peroxide for 15 min. The slides were then incubated with the primary antibody for 2 h in a humidified chamber at room temperature. The detection system used was the Envision-Dual Link System-HRP (Dako, Carpinteria, CA, USA), following the manufacturer’s protocol, with chromogenic substrate staining using 3,3’-diaminobenzidine (Dako, Carpinteria, CA, USA) for 5 min. Counterstaining was performed with Carazzi’s hematoxylin. Positive and negative controls were included in all analyses. Detailed information on the source, antigen retrieval solution, dilution, and clone for each primary antibody is provided in Supplementary Table 2.

Subsequently, the slides were digitized and analyzed using QuPath software. For SMA, CD4, and CD8, the total number of positive stromal cells in 5 to 6 different fields of interest was counted, with each field measuring 0.25 mm^2^, totaling 5 to 6 areas of 0.25 mm^2^ per slide. For CD31 and CD34, the number of positive vessels was counted in 5 to 6 different fields of interest, also measuring 0.25 mm^2^ each. For Vimentin and Claudin-1, the total number of positive neoplastic cells was recorded in 5 to 6 fields of interest, each measuring 0.25 mm^2^ and the intensity of expression was classified as weak (+), moderate (+ +), or strong (+ + +). Finally, for Ki-67 expression analysis, all tumor islands present on the histological slide were selected using the “brush” tool, and the percentage of negative and positive cells was calculated.

### Data analysis

GraphPad Prism software, version 8.0.1 (244), was used for statistical analysis, where means were calculated and compared between groups. One-way ANOVA was used to detect significant differences between group means, and Sidak’s post-hoc test was applied to identify which specific groups presented different means. The significance level was set at less than 0.05 (*p* < 0.05).

## Results

### Histopathological analysis (H&E staining)

**Fig. 1:**
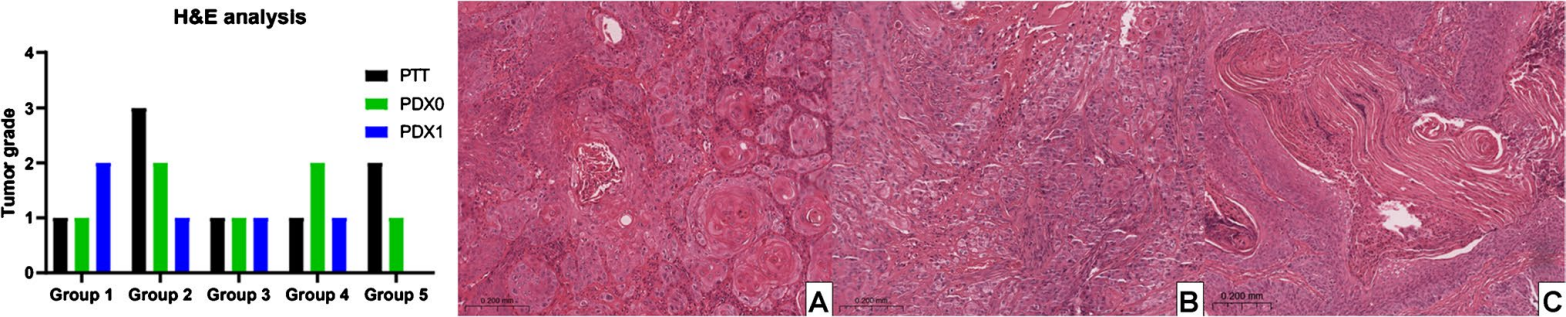
Tumor grade and representative histological panel of patient-derived tumor (PTT), patient-derived xenograft tumor passage 0 (PDX0), and passage 1 (PDX1). **A** H&E stain PTT. **B** H&E stain PDX0. **C** H&E stain PDX1

The tumor grade remained the same between the patient and PDX0 in two cases. In another two cases, there was a decrease in tumor grade in PDX0 compared to the patient’s tumor, and in one case, PDX0 showed an increase in grade compared to the patient’s tumor (Fig. 1, Table 1). When comparing PDX0 with PDX1, there was a decrease in tumor grade in two cases, an increase in one case, and stability in another (Fig. 1, Table 1). In the case of patient number 5, curiously, PDX0 presented a low grade of the tumor, and the implantation of PDX1 was not successful.

**Table 1:** Histopathological information

	Anneroth score*/histopathological grade*		
	PTT	PDX0	PDX1
**Case 1**	5/I	4/I	7/II
**Case 2**	10/III	8/II	5/I
**Case 3**	6/I	4/I	6/I
**Case 4**	6/I	8/II	4/I
**Case 5**	7/II	3/I	NA
	**Histological changes between passages (%)**		
**PTT-PDX0**	3 of 5 cases (60%)		
**PDX0-PDX1**	3 of 4 cases (75%)		

^*^Based on the criteria of Anneroth et al., 1987, *NA* not available

Statistical analyses were not performed due to the limited number of samples; however, the histopathological grade changed in 60% of passages between PTT tumors and PDX0 and in 75% of passages between PDX0 and PDX1, indicating limited preservation of tumor characteristics and histological grade across passages (Table 1).

### Immunohistochemical analysis

**Fig. 2:**
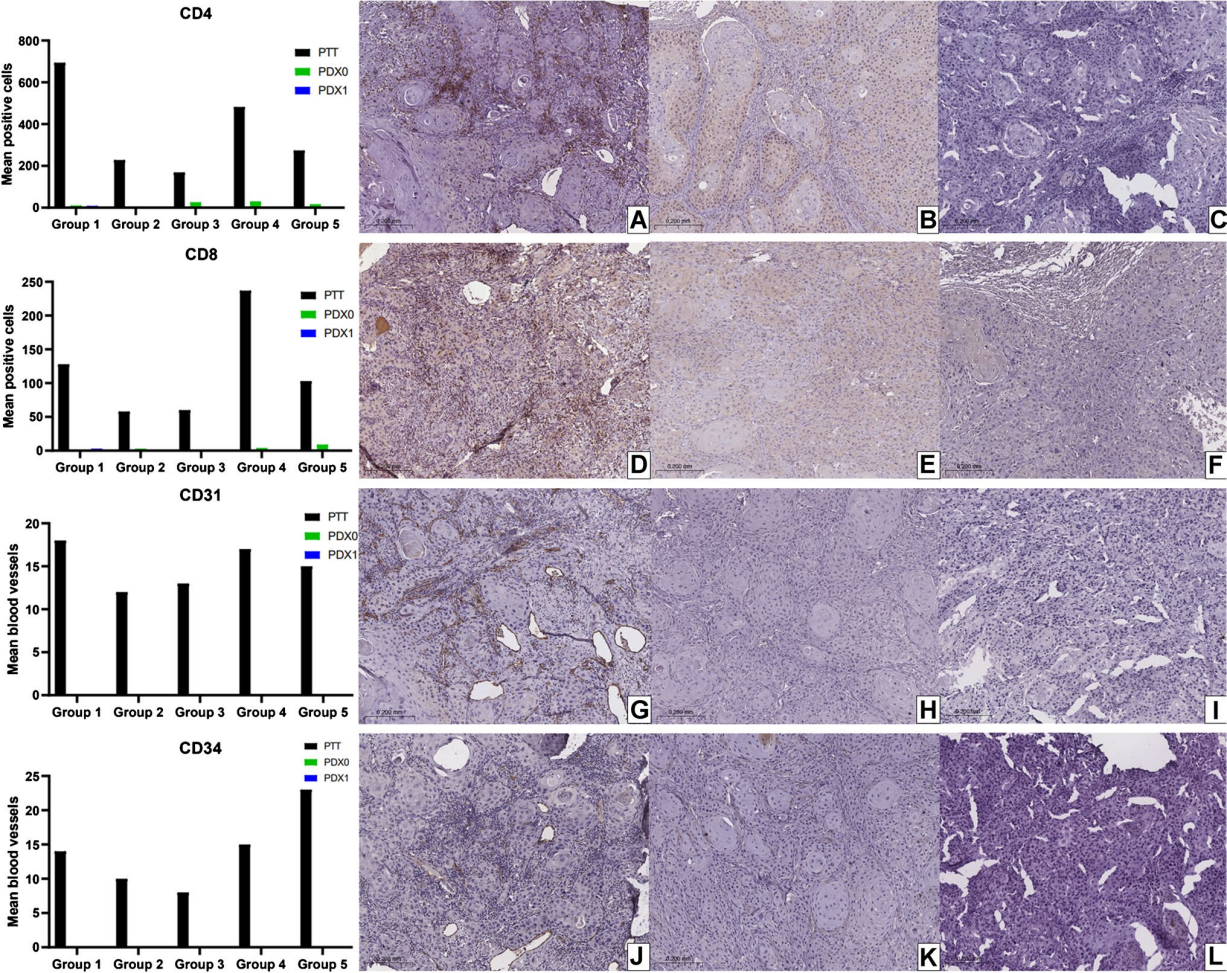
Expression means and representative immunohistochemical panel of patient-derived tumor (PTT), patient-derived xenograft tumor passage 0 (PDX0), and passage 1 (PDX1). CD4. **A** PTT. **B** PDX0. **C** PDX1. CD8. **D** PTT. **E** PDX0. **F** PDX1. CD31. **G** PTT. **H** PDX0. **I** PDX1. CD34. **J** PTT. **K** PDX0. **L** PDX1

Quantification of CD4, CD8, CD31, and CD34 expression revealed a significantly higher mean expression in PTT tumors (Table 2, Fig. 2), compared to PDX0 and PDX1 (Table 2, Fig. 2, Supplementary Fig. 2).

**Table 2:** Immunohistochemical information

	Mean of positive cells/blood vessels**/percent ***	One-way ANOVA *p* value (*p* <0.05)	Sidak’s multiple comparisons test P value (*p* < 0.05)	
			PPT-PDX0	PDX0-PDX1
**CD4**				
PTT	369.4	0.02*	0.04*	0.08
PDX0	18.00			
PDX1	1.8			
**CD8**				
PTT	117.2	0.02*	0.04*	0.50
PDX0	3.2			
PDX1	0.8			
**CD31**				
PTT	15.00	0.00*	*	-
PDX0	0.00			
PDX1	0.00			
**CD34**				
PTT	14.00	0.00*	*	-
PDX0	0.00			
PDX1	0.00			
**SMA**				
PTT	445.8	0.27	0.46	0.39
PDX0	638.2			
PDX1	432.0			
**Claudin-1**				
PTT	151.8	0.26	0.99	0.51
PDX0	146.2			
PDX1	461.8			
**Vimentin**				
PTT	137.6	0.34	0.57	0.57
PDX0	477.6			
PDX1	225.8			
**Ki-67**				
PTT	15.40	0.8210	0.9941	0.7956
PDX0	16.00			
PDX1	13.20			

^*^Statistically significant difference; **for CD31 and CD34; ***for Ki-67; *PTT* primary patient tumors; *PDX0* Model passage 0; *PDX1* model passage 1

**Fig. 3:**
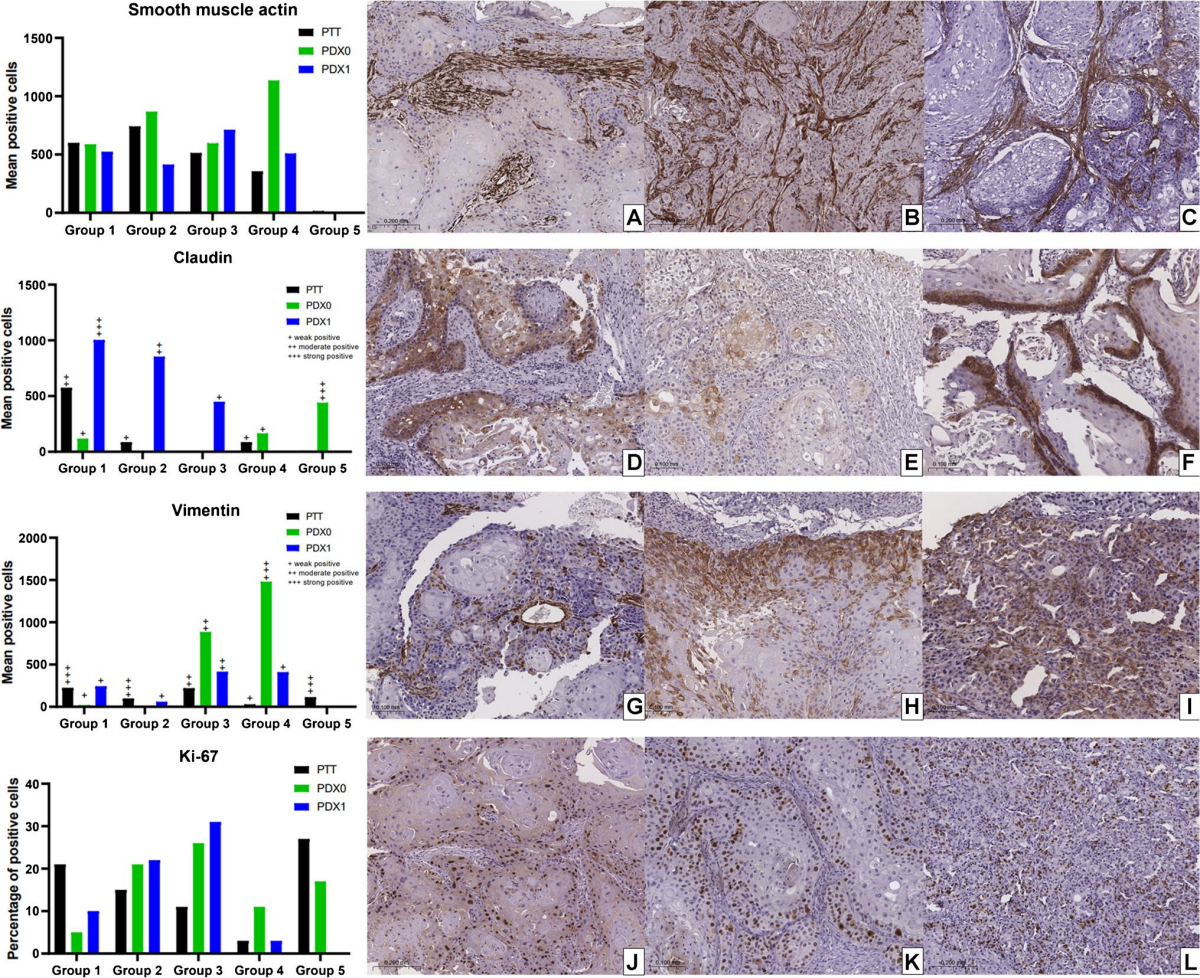
Expression means and representative immunohistochemical panel of patient-derived tumor (PTT), patient-derived xenograft tumor passage 0 (PDX0), and passage 1 (PDX1). SMA. **A** PTT. **B** PDX0. **C** PDX1. Claudin-1. **D** PTT. **E** PDX0. **F** PDX1. Vimentin. **G** PTT. **H** PDX0. **I** PDX1. Ki-67. **J** PTT. **K** PDX0. **L** PDX1

CAFs were investigated using SMA. A similar mean of expression was observed in PTT, PDX0, and PDX1 tumors, with no statistical differences between PTT and PDX0 or between PDX0 and PDX1 (Table 2, Fig. 3, Supplementary Fig. 2).

Expression levels of Claudin-1, Vimentin, and Ki-67 were assessed in neoplastic cells. Differences in mean expression were observed among PTT, PDX0, and PDX1 tumors; however, statistical significance was not achieved (Table 2, Fig. 3, Supplementary Fig. 2).

## Discussion

Tumor induction by PDX involves the implantation of a fragment of cancerous tissue derived from a patient into immunocompromised mice [14, 18]. In a systematic literature review, Schuch et al. [5] reported that several studies have used this model and that the technique recapitulates the histology and molecular features of the original tumors, and also retain the genomic and molecular signatures of the donor-patient [19]. In this study, OSCC PDX tumors preserved CAFs, TME characteristics, and proliferative activity across passages, while exhibiting reduced immune infiltration, loss of tumor-derived vascularization, and variation/instability in histopathological grade between passages.

The TME is a complex ecosystem, comprising diverse cellular and non-cellular elements, such as stromal cells, extracellular matrix (ECM), vasculature, and immune infiltrates, together with neoplastic cells that display marked heterogeneity [20]. Immunohistochemical reactions can aid in identifying different cell populations and confirming histological and population fidelity between PDX tumors and the donor tumor [4], as well as facilitating the understanding of TME components. This motivated us, in the present study, to evaluate, through H&E staining and immunohistochemical reactions, whether PDX tumors maintained the histological characteristics and protein expression patterns of donor tumors across passages, in order to characterize the TME and assess the maintenance of such stromal and neoplastic components.

During conventional histological analysis, we observed that the tumor grade of PDX tumors varied across passages from PTT, PDX0, and PDX1 samples, indicating potential instability or progression of tumor grade between passages and suggesting that this approach preserves similar pathological and architectural features in only a subset of cases. Interestingly, in case 5, the PDX0 tumor developed cystic areas, in contrast to the PTT tumor, which prevented implantation and establishment of PDX1. Our findings differ from those of Swick et al. [21] and Li et al. [22], who reported that histological similarity was maintained in their studies involving OSCC-derived PDX tumors; however, this discrepancy may be explained by methodological differences and the analytical approaches employed across studies.

Pearson et al. [2] also employed this methodology to establish PDX tumors for head and neck squamous cell carcinoma (HNSCC) and adenoid cystic carcinoma (ACC). Similar to our findings, HNSCC models showed increased histological grade (nuclear pleomorphism and a decreased stromal proportion) across successive passages. Although passages were performed more frequently by Pearson et al. [2], we likewise observed histological grade progression in some cases, which, according to Pearson et al. [2], may increase with a higher number of passages. In addition, the authors emphasize the importance of better understanding whether the clinical aggressiveness of the tumor can influence histological grade, beyond the faster growth observed in PDX tumors. In our study, it is worth noting that among the five cases, two were well differentiated, two were moderately differentiated, and one was poorly differentiated, which may influence the degree of histological differentiation observed in PDX tumors. For morphological classification and comparison between groups, we adopted the criteria described by Anneroth et al. [17], which provide a standardized morphological assessment across the entire tumor. Other classification systems, such as that proposed by Bryne et al. [23], focus exclusively on features at the deepest invasive margins; however, this approach was not feasible for all cases in our series, and in PDX tumors, capsule formation in some cases may further hinder this analysis.

We next used immunohistochemistry to characterize TME components and neoplastic cells’ features. CAFs are critical components of the TME, as they secrete growth factors, inflammatory ligands, and extracellular matrix proteins that are associated with tumor proliferation and resistance to anticancer treatments [24]. Therefore, they may play a role in treatment resistance and tumor progression [24–26]. Alpha-SMA marker has been used to detect this cell population, including in OSCC cases [27]. Our results showed that CAFs’ presence remained stable in PDX tumors, with no significant differences between passages and their tumors of origin, indicating that this model is suitable for future studies focusing on this cell population within the TME component. Notably, CAF-targeting strategies are increasingly being explored across various cancer types, including OSCC [28]. In line with this, a recent study using breast cancer PDX models demonstrated that CAF subtypes can directly influence treatment response: in paclitaxel-resistant triple-negative breast cancer, tumors exhibited an enrichment of cycling CAFs, fibroblasts with active cell cycle features, alongside JAK/STAT pathway dysregulation. Targeting JAK1/2 not only restored paclitaxel sensitivity in some resistant models but also altered CAF subtype composition, underscoring the potential of PDX systems for dissecting CAF-driven mechanisms of therapy resistance [29].

Beyond stromal components such as CAFs, immune cells represent another key element of the TME, contributing to both tumor control and progression. Helper and cytotoxic T cells were investigated using CD4 and CD8 markers. These markers were minimally or not detected in PDX tumors, despite their presence in the corresponding PPT. This reduction likely reflects the inability to sustain these immune cell populations after tumor implantation, a phenomenon linked to one of the most relevant limitations of PDX models, the immunosuppressed status of the host animals [30]. Notably, such immunosuppression is essential to ensure tumor engraftment in this system [31]. Tumor-infiltrating lymphocytes, however, remain integral components of the TME and can mediate either antitumor immunity or immune suppression [32, 33]. In OSCC, the presence of CD3⁺, CD4⁺, and CD8⁺ tumor-infiltrating lymphocytes (TILs) has been associated with patient prognosis, with low CD4⁺ expression in early-stage disease identifying a subgroup with survival outcomes comparable to advanced-stage cases [34]. In HNSCC, patients with PD-L1–low/CD8⁺ TIL–high tumors demonstrated no local failure or disease-related death following radiotherapy, highlighting the prognostic and potentially predictive value of TILs in treatment response [35]. Consequently, the immune contexture of PDX tumors may influence both their intrinsic biology and their responsiveness to experimental treatments, particularly in studies targeting TME components in OSCC.

Tumor growth and development require vascular formation to supply oxygen and remove metabolites; additionally, tumor growth generates a hypoxic environment, which triggers angiogenesis [36, 37]. With the formation of blood vessels supporting tumor growth, tumor cells can enter the bloodstream and cause distant metastases [38]. Moreover, normalizing vascularization within the TME is crucial to increase T cell infiltration into the tumor, in addition to supporting studies on immunotherapy and anti-angiogenic therapies [39]. The presence and importance of blood vessels in the TME are well understood. Our results suggest that PDX tumors showed a significantly lower number of CD31 and CD34-positive vessels compared to PTT tumors, indicating that revascularization occurs with endothelial cells from the host animal after tumor implantation, demonstrating interaction between the TME and the animal. Furthermore, these findings suggest that, since there is no vascular recanalization but rather revascularization by the host animals, such events may hinder the successful establishment of tumors in some cases, which could explain the success rate of approximately 50% [5].

Epithelial-mesenchymal transition is a change in epithelial cells, in which they shift from an immobile epithelial phenotype to a mobile mesenchymal phenotype [40], characterized by the loss of intercellular junction proteins typically present in epithelial cells. Changes may occur in cell membrane proteins, such as Claudin-1, which maintains intercellular adhesion and prevents the release of neoplastic cells to distant sites [41]. Additionally, during epithelial-mesenchymal transition, neoplastic cells acquire the expression of mesenchymal proteins, such as vimentin [42]. Thus, in malignant neoplasms, the expression of Claudin-1 and vimentin confers invasive characteristics, enhancing metastasis potential [43]. The mean expression levels of Claudin-1 and vimentin were similar between PTT and PDX0 tumors, as well as between PDX0 and PDX1, with no statistical differences, indicating that epithelial-mesenchymal transition features are maintained across passages, faithfully preserving these characteristics from the original tumor.

The Ki-67 marker is positively expressed during various stages of the cell cycle (G1, S, G2, and M), and it reflects the biological behavior of tumors since it is a nuclear protein associated with cell proliferation. This marker is widely used in immunohistochemistry to assess the level of cell proliferation in tumors, including OSCC [44]. In our results with this marker, we observed that the mean expression levels were similar between PTT and PDX0 tumors, as well as between PDX0 and PDX1, with no significant differences, indicating that this model supports tumor progression. Chen et al. [45], when implementing PDX tumors of osteosarcoma in mice, also reported that histology and Ki-67 expression were similar between PDX tumors and donor tumors from patients; however, as discussed above, if a greater number of passages are performed, the tumor grade may increase, as observed by Pearson et al. [2], and in this scenario, the increase in Ki-67 expression could increase critically.

We acknowledge several limitations of this study, primarily related to the sample size and the number of passages, as we present comparative data from five cases between PTT, PDX0, and PDX1. This limited certain statistical analyses; therefore, p-values should be interpreted with caution. This limitation was imposed by ethical considerations. Nevertheless, this is the first study to comprehensively describe the characteristics of the TME in PDX tumors, comparing them with their respective original tumors. A second limitation concerns histological grade classification, as we observed fluctuations across passages in more than 50% of the samples; however, an ideal statistical analysis could not be performed due to the limited number of samples to determine whether these differences were significant. The use of alternative grading systems could provide additional insights; however, many contemporary approaches rely on morphological features at the tumor invasive front, such as invasion pattern or tumor budding [46–48], which are often difficult to identify or are not reliably preserved in PDX models. This makes these systems less suitable for our dataset. The Anneroth system [17] was therefore selected because it provides a consistent assessment across the entire tumor area and is more feasible in the context of PDX morphology. Finally, we highlight that other biomarkers of potential interest for TME evaluation were not included in our analyses, nor were molecular analyses performed, which could have been useful for a more in-depth comparison between PDX and PTT tumors.

In conclusion, OSCC PDX tumors may exhibit instability or an increase in histopathological grade across passages. Regarding tumor and TME components, we observed that the mean expression levels of SMA, Claudin-1, Vimentin, and Ki-67 were preserved, indicating the maintenance of CAFs and the characteristics of epithelial-mesenchymal transition and tumor proliferation even after two serial passages. The presence of CD4 and CD8 inflammatory cells was significantly reduced in PDX tumors, and the positivity of blood vessels for CD31 and CD34 was lost in PDX tumors, indicating the absence of inflammatory cells and that tumor revascularization occurs through host-derived cells. These findings are important for guiding future studies testing treatments involving the TME in OSCC-derived PDX tumors, especially in therapies targeting inflammatory cells and vascularization.

## Supplementary Information

Below is the link to the electronic supplementary material.

**Supplementary Fig. 1** Methodological scheme for implementation of patient-derived xenograft tumors and analysis of tumor microenvironment components (JPEG 43.9 KB)

**Supplementary Fig. 2** Confidence intervals of the Sidak statistical test(PNG 1.95 MB)

High Resolution Image (TIF 549 KB)

**Supplementary Table 1** Patient clinical information, tumor differentiation grade, and TNM classification (DOCX 21.7 KB)

**Supplementary Table 2** Primary antibodies used for the immunohistochemical staining (DOCX 14.7 KB)

## Data Availability

All data generated by this study are available in this article and its supplementary materials. Additional information is available from the corresponding author upon reasonable request.
