# Supplementary figures and images for: Can oral squamous cell carcinoma xenografts tumors mirror the original tumor microenvironment? An immunohistochemical analysis
Source: Virchows Arch. 2026 Jan 27;488(3):627–37. doi: 10.1007/s00428-026-04399-0 (PMC12963117; doi:10.1007/s00428-026-04399-0)

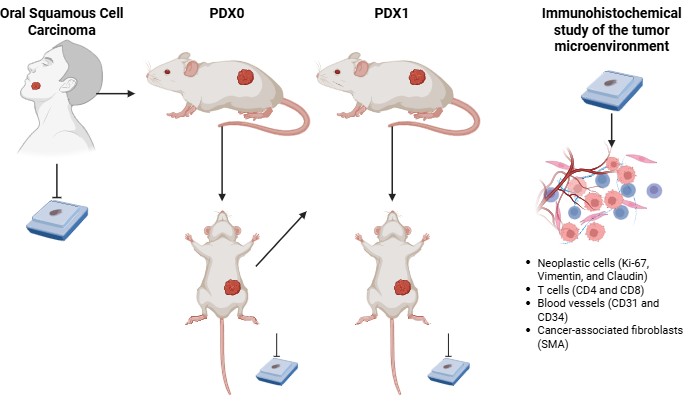

Supplement: Supplementary file 1 — Supplementary Fig. 1 Methodological scheme for implementation of patient-derived xenograft tumors and analysis of tumor microenvironment components (JPEG 43.9 KB) [file 428_2026_4399_MOESM1_ESM.jpeg]

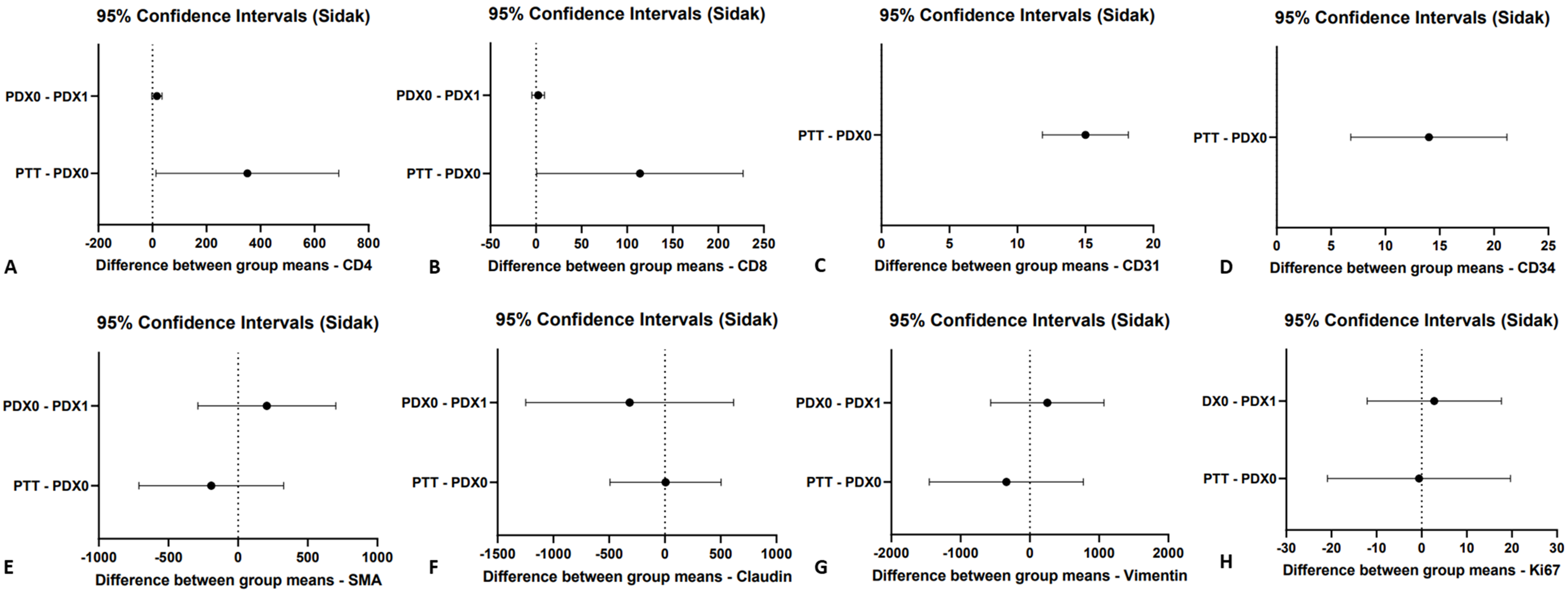

Supplement: Supplementary file 2 — Supplementary Fig. 2 Confidence intervals of the Sidak statistical test(PNG 1.95 MB) [file 428_2026_4399_Fig4_ESM.png]

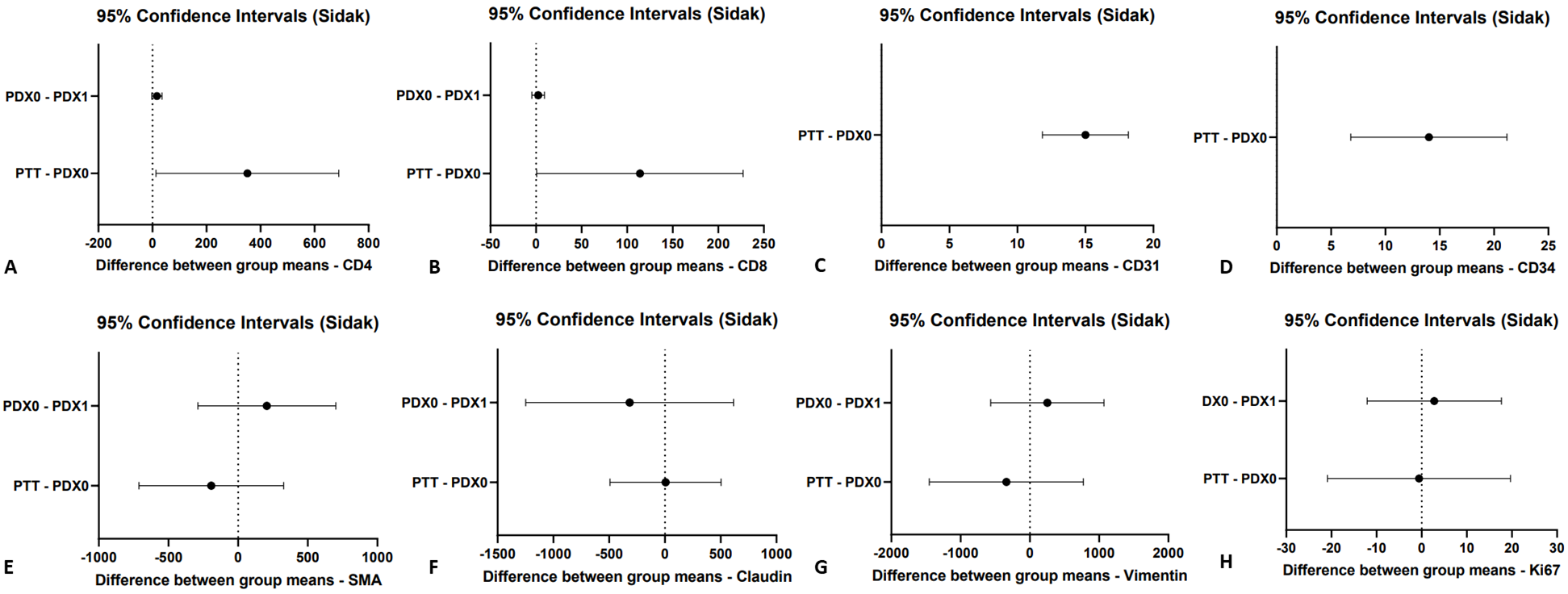

Supplement: Supplementary file 3 — High Resolution Image (TIF 549 KB) [file 428_2026_4399_MOESM2_ESM.tif]
