# Supplementary material for: Can oral squamous cell carcinoma xenografts tumors mirror the original tumor microenvironment? An immunohistochemical analysis
Source: Virchows Arch. 2026 Jan 27;488(3):627–37. doi: 10.1007/s00428-026-04399-0 (PMC12963117; doi:10.1007/s00428-026-04399-0)
Supplement: Supplementary file 4 — Supplementary Table 1 Patient clinical information, tumor differentiation grade, and TNM classification (DOCX 21.7 KB) [file 428_2026_4399_MOESM3_ESM.docx]

**Supplementary Table 1** - Patient clinical information, tumor differentiation grade and TNM classification.

| **Patient number** | **Sex** | **Age** | **Tobacco consumption** | **Alcohol consumption** | **Tumor location** | **Degree of differentiation in surgical speciment** | **TNM** |
| --- | --- | --- | --- | --- | --- | --- | --- |
| 1 | Male | 51 | Yes | Yes | Tongue | Poorly differentiated | T3N1M0 |
| 2 | Male | 69 | Yes | Yes | Floor of the mouth | Moderately differentiated | T3N2aM0 |
| 3 | Male | 54 | Yes | Yes | Tongue | Well differentiated | T3N0M0 |
| 4 | Male | 63 | Yes | Yes | Tongue | Moderately differentiated | T3N2cM0 |
| 5 | Male | 62 | Yes | Yes | Floor of the mouth | Well differentiated | T3N0M0 |
