# Supplementary material for: Can oral squamous cell carcinoma xenografts tumors mirror the original tumor microenvironment? An immunohistochemical analysis
Source: Virchows Arch. 2026 Jan 27;488(3):627–37. doi: 10.1007/s00428-026-04399-0 (PMC12963117; doi:10.1007/s00428-026-04399-0)
Supplement: Supplementary file 5 — Supplementary Table 2 Primary antibodies used for the immunohistochemical staining (DOCX 14.7 KB) [file 428_2026_4399_MOESM4_ESM.docx]

**Supplementary Table 2** - Primary antibodies used for the immunohistochemical reactions.

| **Antibody** | **Clone** | **Source** | **Antigen Retrieval** | **Dilution** |
| --- | --- | --- | --- | --- |
| SMA | 1A4 | Dako | Citrate | 1:300 |
| CD4 | SP35 | Spring bioscience | Citrate | 1:50 |
| CD8 | C8/144B | Dako | Tris/EDTA | 1:100 |
| CD31 | JC70A | Dako | Tris/EDTA | 1:200 |
| CD34 | QBEnd-10 | Dako | Citrate | 1:100 |
| Claudin-1 | Polyclonal | Diagnostic BioSystems | Citrate | 1:100 |
| Vimentin | V9 | Dako | Citrate | Ready-to-use |
| Ki-67 | MIB-1 | Dako | Tris/EDTA | 1:300 |
